# How does authentic leadership support employee resilience? An explanation from cognitive-affective personality system theory

**DOI:** 10.3389/fpsyg.2026.1728062

**Published:** 2026-02-12

**Authors:** Xu Li, Bingrong Li

**Affiliations:** 1 School of Humanities, Tianjin Agricultural University, Tianjin, China; 2 School of Business, Tianjin University of Finance and Economics, Tianjin, China

**Keywords:** authentic leadership, cognitive-affective personality system theory, emloyee vigor, employee resilience, employee traditionality, role breadth self-efficacy

## Abstract

The sustainable development of organizations depends on how employees develop resilience in the face of unexpected crises and changes. Leadership has been proven to have an important impact on employee resilience. Therefore, the paper explores how authentic leadership supports employee resilience through the cognitive-affective perspective. In study 1, the authors conducted a scenario experiment and recruited 247 MBA students to participate. In study 2, the authors collected two-wave data from 424 employees and 85 leaders in China. Findings reveal that authentic leadership effectively enhances employee resilience by strengthening employee vigor and role breadth self-efficacy (RBSE). Besides, employee traditionality is found to negatively moderate the relationship between authentic leadership and employee vigor, whereas its moderating effect on the link between authentic leadership and RBSE is non-significant. This research enriches the literature on employee resilience and provides valuable insights for improving the effectiveness of organizational leadership.

## 1 Introduction

In the current environment of instability and uncertainty, employee resilience is seen as a crucial force for organizations to survive and sustainable development (Lengnick-Hall and Beck, 2005; Näswall et al., 2019; Liu et al., 2024). Employee resilience is portrayed as an adaptive behavioral capacity of employees, with which, one can utilize resources and seek out opportunities to deal with stress and challenges in the workplace, and even achieve continuous improvement, especially in a crisis and uncertain context (Kuntz et al., 2016; Liu et al., 2024). Research has consistently demonstrated that employee resilience, as a critical competitive advantage for organizations, not only exerts positive effects on job satisfaction (Liu et al., 2024), job wellbeing (Iqbal and Piwowar-Sulej, 2022), and employee engagement (Cooke et al., 2019), but also contributes to enhance organizational commitment (Karatepe and Karadas, 2014), improve organizational performance (Li and Zhang, 2022), reduce turnover intention (Mao et al., 2022), and even ensure organizational sustainability (Carnevale and Hatak, 2020). Given this recognized importance, exploring strategies to support employee resilience has emerged as a key focus for both scholars and practitioners.

Researchers have extensively explored the antecedents of employee resilience, such as individual traits (Anser et al., 2022), learning-oriented organizational climate (Caniëls and Baaten, 2019), social support (Todt et al., 2018), human resource management (Lu et al., 2022; Shao et al., 2023), and social media usage (Ma et al., 2025). They also found that leadership as a key player in the organizational environment is an important motivating factor that cannot be ignored (Zhu et al., 2019; Djourova et al., 2020; Li and Hu, 2025). Authentic leadership is described as being open to diverse suggestions, presenting authentic selves to subordinates, and holding firm to their moral standards, which are particularly relevant under uncertainty (Todt et al., 2019; Li and Zhang, 2022). Prior studies have offered valuable insights in how authentic leadership improves employee resilience (Rego et al., 2012). Thus, far, the existing studies predominantly focus on employees' psychological states or cognitive processes as the mediator (Li and Zhang, 2022; Mao et al., 2022), ignoring the emotional factors of employees. The relationship between leaders and employees is not merely a contractual exchange, but also intertwined with complex emotional (Zheng et al., 2023) Existing literature also indicates that emotions and cognition are two core factors that jointly drive employee attitude or behavior (Lee and Allen, 2002; Zhao and Ma, 2025). Hence, drawing on cognitive-affective personality system (CAPS) theory, this study aims to unpack the relationship between authentic leadership and employee resilience from an integrative dual lens of cognition and affection, thereby addressing the limitations of previous research.

When facing uncertain and challenging circumstances, employees are required to carry out additional work activities beyond conventional requirements (Raub and Liao, 2012; Kang et al., 2022). Role breadth self-efficacy (RBSE) reflects an employee's confidence in executing a broader set of proactive and challenging work tasks beyond conventionally established standards (Parker, 1998; Kang et al., 2022), serving as a crucial cognitive foundation for fostering employee resilience. Meanwhile, authentic leadership provides employees with trust and support and encourages open communication, which enrich cognitive resources of employees, and motivate them to continuously explore new knowledge and skills (Zheng et al., 2023), thereby enhancing their RBSE. Hence, from the perspective of cognitive path, we explore the mediating role of RBSE. From the perspective of affective path, employee vigor refers to a positive emotional state in which employees feel energetic and passionate at work, playing a crucial role in helping employees cope with challenges and strive to achieve work goals (Shirom, 2011; Gao et al., 2020; Liu et al., 2024). And authentic leadership emphasizes positive work climate and high-quality interpersonal relationships, which can effectively reduce career burnout and enhance employee vigor (Srimongkolkul et al., 2025). Therefore, we explore employee vigor as affective mediator.

Furthermore, authentic leadership is not always effective (Zheng et al., 2022). The literature has consistently demonstrated that the relationship between leadership and employee outcomes is contingent upon individual cultural characteristics (Yang et al., 2021; Zheng and Ahmed, 2022). Meanwhile, CAPS points out that individual differences could serve as a key explanatory mechanism linking external situational contexts to individuals' cognitive-affective responses (Yuan et al., 2019). Employee traditionality, as one of the most critical cultural value variables in Asian contexts, captures one's endorsement of traditional hierarchical role relationships and plays an important role in the impact and effectiveness of leadership (Farh et al., 2007; Xu et al., 2024). However, little is known about the role of employee traditionality in influencing authentic leadership process. To address this gap, we explore the moderating role of employee traditionality in the cognitive (RBSE) and affective (employee vigor) paths through which authentic leadership influences employee resilience.

**Figure 1:**
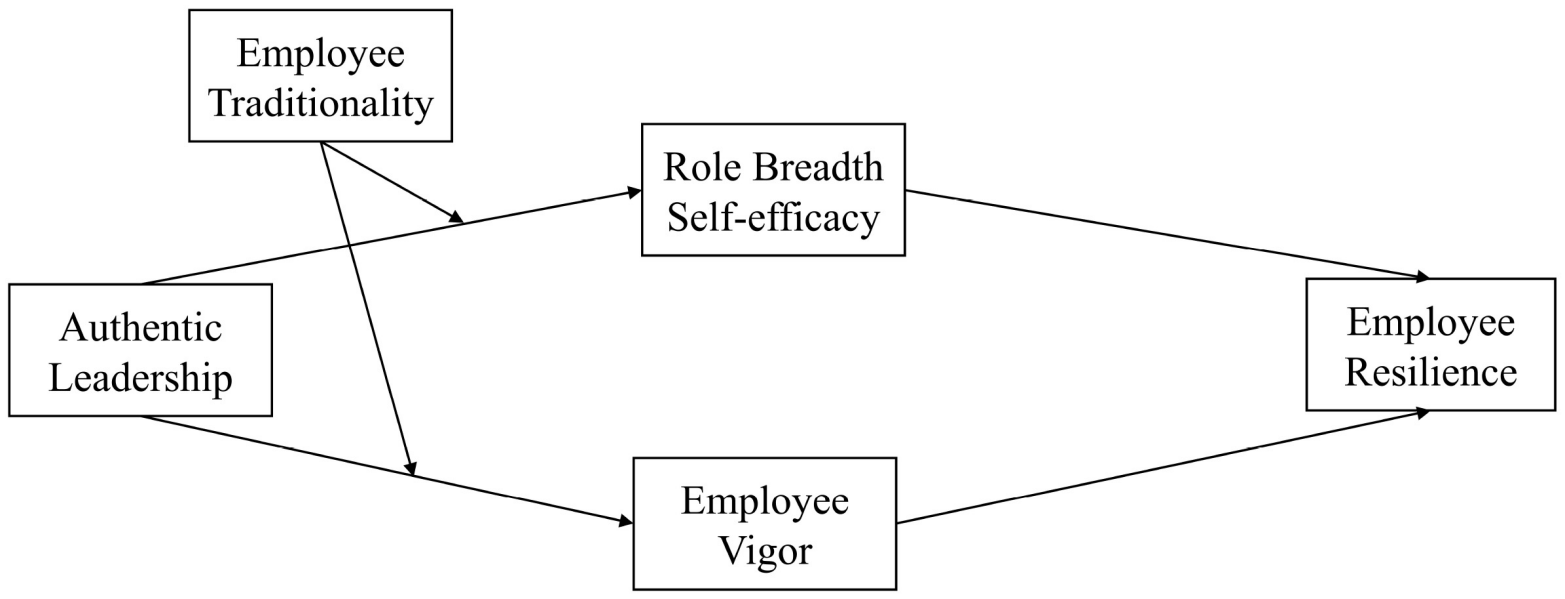
Theoretical model.

By examining these perspectives, this study advances three primary theoretical contributions. First, it uncovers the dual-path mediating mechanism of cognition and affection that link authentic leadership and employee resilience, addressing the limitations of a single perspective in prior research and providing a new research perspective for exploring the influence mechanism of employee resilience. Second, this research explores the moderating influence of employee traditionality on dual path, not only directly responding to recent calls for examining cultural factors in the leadership process (Cheng et al., 2021), but also describing the conditions under which authentic leadership could lead to positive internal states and strengthen employee resilience. Finally, grounded in CAPS theory, this study develops a more systematic and comprehensive theoretical framework to explicate how authentic leadership supports employee resilience, thereby expanding the application range of CAPS and providing guidance for organizations to help employees cope with challenges and enhance resilience in practical work. The theoretical model of this paper is shown in Figure 1.

## 2 Theoretical background and hypotheses

### 2.1 Cognitive-Affective Personality System (CAPS) Theory

Developed by Mischel and Shoda, 1995, CAPS emphasizes the fact that the generation of individual responses and behaviors is determined by the dynamic interactions between cognitive appraisals and affective responses triggered by situational cues. Upon confrontation with specific events or situations, individuals' complex cognitive-affective units will be activated and ultimately lead to the resulting responses or behaviors (Yuan et al., 2019; Fan et al., 2025). Compared to social exchange theory, which emphasizes reciprocity and resource exchange (Cropanzano and Mitchell, 2005) and social information processing theory, which focuses on the perception and understanding (Salancik and Pfeffer, 1978), CAPS relates more directly to an individual's internal variability and context sensitivity, offering a process-oriented lens for understanding response or behavior formation (Mischel and Shoda, 1995; Su et al., 2025).

Nowadays, CAPS has been widely applied in fields such as applied psychology, organizational behavior, and consumer behavior to examine complex behavioral mechanisms. For example, Fan et al. (2025) investigated the influence mechanism of role stress on work engagement via both cognitive and affective pathways (organization-based self-esteem and work alienation) based on CAPS. Zheng et al. (2023) applied CAPS to explore how transformational and transactional leadership styles influence employees' pro-environmental behaviors through activating a work promotion/prevention focus (cognitive path) and modulating emotional exhaustion (affective path). Likewise, in consumer behavior, CAPS highlights the dynamic interplay between emotional resonance and cognitive rationality in consumer decision-making (Su et al., 2025; Zhou et al., 2025). These cross-domain applications underscore the utility of CAPS as a universal framework for analyzing response or behavior formation.

Hence, from the perspective of CAPS, we argue that authentic leadership, as an external situational factor, could activate employees' cognitive and emotional states, which in turn influence their resilience. Further, CAPS provides an integrated cognitive and affective perspective, which will help to comprehensively reveal the process of the impact of authentic leadership on employee resilience through the individual's complex internal mechanisms.

### 2.2 Authentic leadership and employee resilience

Authentic leadership is regarded as a positive leadership style, which encompasses four core dimensions: self-awareness, internalized moral perspective, balanced processing, and relational transparency (Zheng et al., 2022; He et al., 2025). These dimensions collectively foster the cultivation of a positive, ethical, and supportive organizational climate (Avolio et al., 2004; Walumbwa et al., 2008). Self-awareness refers to a leader's capacity to recognize their own strengths and weaknesses, as well as the impact they exert on their followers (Zheng et al., 2022). Balanced processing involves being open to diverse opinions, and systematically and impartially evaluating all relevant information prior to finalizing decisions. Relational transparency means that leaders could present their authentic feelings and opinions with subordinates (Zheng et al., 2022). Internalized moral perspective is characterized by a firm commitment to upholding high moral standards (He et al., 2025).

Leadership, recognized as a critical contextual factor, plays a pivotal role in shaping employees' responses to stressful and challenging workplace environments (Wang et al., 2018). Hence, leadership is also considered to be an important trigger for employee resilience (Li and Zhang, 2022). Authentic leadership stays open to diverse opinions, present their true feelings and hold fast to their own internal moral standards, which lay the foundation for employees to response to the challenging environment (Todt et al., 2019). Moreover, authentic leadership is increasingly hailed as a pivotal catalyst for organizational performance, particularly within dynamic and challenging environments (Alvesson and Sveningsson, 2013). Based on previous research, we propose that authentic leadership can significantly support employee resilience. Specifically, first, authentic leaders exhibiting self-awareness make their employees engage in similar introspection, thereby gaining clarity about their own identity (Walumbwa et al., 2008). In the face of challenges or difficulties, employees can utilize their strengths to solve problems. Second, authentic leaders' internalized moral perspective conveys integrity and consistency rather than external pressures (He et al., 2025), which can help employees maintain their integrity when facing difficulties, strive to adapt to the environment and challenges, and achieve positive outcomes. Third, authentic leaders' relational transparency presents the authentic self and true inner thoughts to subordinates. Simultaneously, the balanced processing of authentic leadership create an inclusive climate in which employees can express opinions and even propose challenging viewpoints different from other team members. This integration of leadership behaviors not only enables subordinates to develop a profound sense of trust and psychological safety (Gill and Caza, 2018), but also supports them to drive organizational improvement (Burris, 2012). Employees are more inclined to proactively engage in learning and openly share insights and innovative ideas (Boekhorst, 2015). As a result, when confronted with challenges, employees are more inclined to proactively pursue innovative solutions, which fortifies and enhance employee resilience. This leads to the following hypothesis:

*H1*. Authentic leadership positively affects employee resilience.

### 2.3 The mediating role of RBSE

Role breadth self-efficacy (RBSE), as a specific form of self-efficacy, is described as the degree to which individuals are confident in their ability to perform a range of proactive, integrative, and interpersonal tasks beyond formally prescribed technical requirements (Parker, 1998; Schaubroeck et al., 2017). Different from self-efficacy focusing on a specific task, RBSE emphasizes individual's confidence in regarding the ability to successfully work in a broader array of areas (Hao et al., 2018). Individuals with high RBSE could take on new roles, successfully execute innovative and challenging tasks (Hong et al., 2016) and advance organizational objectives (Schaubroeck et al., 2017).

Grounded in CAPS theory, external situational factors can activate cognitive-affective processes within individuals, which thereby shape behavioral outcomes (Mischel and Shoda, 1995). As a cognitive variable, RBSE is also an important mediating variable that transfers the influence of external situational factors to individual behaviors. Wu and Parker, 2017 reported that leadership, as an important organizational context factor, has a significant impact on employee RBSE. Strauss et al. (2009) also suggested that the supports from leaders enhance the confidence of employees to develop extra-role behaviors and take on new roles, thus promote the development of high-level employee RBSE. Hence, we suggest that authentic leadership could effectively enhance employee RBSE. Specifically, authentic leaders foster the open exchange of critical information, promote the authentic expression of personal perspectives and emotions, and cultivate a receptive environment for diverse viewpoints (Li and Zhang, 2022; Zheng et al., 2022). Such behaviors signal to the employees that the organization encourages mutual learning and innovation, and view failure and difficulties positively. In this kind of context, employees feel comfortable, safety and supportive (Farnese et al., 2019). Consequently, they become more confident in their abilities to multitask, and tend to carry out exploratory activities, propose creative ideas, and take initiative at work (Schaubroeck et al., 2017), which promote employee RBSE.

According to CAPS theory, when an individual's cognitive unit is activated, it will result in the corresponding behavior of the individual through a series of chain reactions (Mischel and Shoda, 1995). RBSE, which reflects individuals' positive psychological cognition plays a pivotal role in employee resistance to pressure, challenges and difficulties. Specifically, employees with high RBSE demonstrate stronger confidence in their capability to effectively execute tasks beyond their formal job requirements and engage in extra-role behaviors (Galperin, 2012). When encountering difficulties or failures in their work, employees with high RBSE also have a stronger resistance to setbacks and are more convinced of their abilities to navigate diverse challenges and difficulties (Den Hartog and Belschak, 2012). This positive cognition promotes employees to adapt to environment more quickly, and proactively engage in learning and find solutions to problems, even beyond the prescribed work requirements, and complete multiple tasks (Kang et al., 2022). They are also more likely to take on challenging tasks instead of avoiding them (Beltrán-Martín et al., 2017; Shao et al., 2025). All of these support employee resilience. Based on the above reasoning, RBSE provides positive psychological cognitive support for employees. Authentic leaders may promote employee resilience through influencing employee RBSE (cognitive unit). Based on this reasoning, the present study proposes the following hypothesis:

*H2*. The positive relationship between authentic leadership and employee resilience is mediated by RBSE.

### 2.4 The mediating role of employee vigor

According to CAPS theory, individual affective response to external situations, is an important factor leading to individual attitude or behaviors (Mischel and Shoda, 1995). Employee vigor is described as “a set of positive and energetic affective states that individuals experience at work” (Kark and Carmeli, 2009; Liu et al., 2024). Research indicates that positive work climate and high-quality interpersonal relationships in the workplace are critical drivers in invigorating employees (Spreitzer et al., 2005). As the “key person” and “atmosphere engineer” in the organization, effective leadership plays a vital role on shaping the organizational atmosphere and cultivating good relationships (Carmeli et al., 2009). Given the instrumental role of authentic leaders in cultivating an open and transparent organizational climate (Zheng et al., 2022; He et al., 2025), we contend that authentic leadership exerts a positive effect on nurturing employee vigor. First, authentic leaders' self-awareness, could deeply understand themselves and their subordinates, even receive feedback from others, which fosters a positive and developmental relationship with their followers (Alavi and Gill, 2017). As a result, employees feel supported by leaders and then their positive emotions and work vigor are stimulated. Second, authentic leaders, driven by balanced processing and relational transparency, encourage collaboration and open communication, which foster an open, trustful and enabling work environment (Farnese et al., 2019). In this kind of positive context, employees not only can keep energetic and in good working condition, but also learn from each other actively, which enhance their ability and activity of thinking (Cai et al., 2020).

CAPS posits that positive emotion constitutes a critical component of the mechanism through which external situations shape individual behavior (Mischel and Shoda, 1998). By encoding the situational characteristics, individuals stimulate their own affective units, and then produce explicit attitudes or behaviors. As an affective construct, employee vigor represents the positive emotions and attitudes individuals hold toward their work. Research has consistently linked it to significant individual and organizational outcomes (Shirom, 2009). An individual experiencing vigor are perceived as peppy, physically enlivened, cognitively quick and creative (Little et al., 2011). All of these factors can further motivate employees to recover more swiftly from setbacks (Sonnentag and Fritz, 2007), develop innovative solutions for problems and then energetically fulfill the tasks (Cai et al., 2020). Additionally, vigor at work can also be conceptualized as a positive emotional resource (Shirom, 2009) to make employees less prone to stress, feel more confidence and courageous, and tend to take the initiative to solve problems in the challenging environment (Carmeli et al., 2009; Liu et al., 2024). Based on the preceding reasoning, we contend that vigor at work endows employees with abundant energy, facilitates swifter recovery from setbacks, and even propels proactive engagement in overcoming difficulties and challenges. Ultimately, employee resilience is enhanced. Hence, given that vigor at work provides positive emotional support for employees, we suggest that authentic leadership can support employee resilience through influencing employee vigor (affective unit). Building on the preceding reasoning, the present study proposes the following hypothesis:

*H3*. The positive relationship between authentic leadership and employee resilience is mediated by employee vigor.

### 2.5 The moderating role of employee traditionality

Traditionality, as a typical personality trait, captures the degree to which an individual endorses the traditional values or adheres to the traditional hierarchical role relationships (Farh et al., 2007; Xu et al., 2024). Submission to authority constitutes the core element of employee traditionality within the workplace context (Farh et al., 2007). Employees with high traditionality tend to exhibit unconditional compliance with their leaders and strictly adhere to prescribed role obligations, whereas those with low traditionality are more inclined to follow rules grounded in the balance between incentives and contributions (Farh et al., 1997). Hence, even when interacting with the same leader, employees with varying levels of traditionality may develop distinct interpretations of leadership styles and behaviors, thereby exhibiting divergent attitudes and behavioral patterns (Yao et al., 2020). Prior research has documented the moderating role of employee traditionality in shaping the relationships between leadership and subordinates' performance (Liu et al., 2013; Cheng et al., 2021; Zheng et al., 2022). Furthermore, CAPS theory posits that individual traits can help explain the mechanisms linking external situations to cognitive-affective responses (Yuan et al., 2019). Drawing on these insights, we propose that employee traditionality moderates the positive effect of authentic leadership on both RBSE and employee vigor.

Authentic leaders could promote reciprocal support and shape an open and transparent atmosphere in the workplace (Farnese et al., 2019), which not only reduce formal hierarchical differences, but also emphasize equal communication in the organization environment. However, employees with higher traditionality tend to take hierarchical distinctions between leaders and subordinates for granted, and consider it incumbent upon themselves to adhere to prescribed role obligations (Zheng and Ahmed, 2022). Hence, they are less sensitive to the behaviors of authentic leaders and less likely to be influenced by the way authentic leaders treat them (Cheng et al., 2021). As a result, their cognitive level and emotional state at work are less likely to be promoted by an authentic leader, leading to a weaker relationship between authentic leadership and RBSE as well as employee vigor. Conversely, employees with lower traditionality prioritize equality, fairness, openness, and job autonomy, while exhibiting weaker hierarchical consciousness and a stronger desire for respect and support (Farh et al., 1997; Yang et al., 2021). Authentic leadership cultivates a work environment characterized by openness, support, respect, and equality which satisfies the needs of employees with lower traditionality. These employees identify strongly with authentic leadership behaviors and respond more positively, thereby enhancing both RBSE and employee vigor. Based on this reasoning, the present study proposes the following hypotheses:

*H4*. Employee traditionality moderates the relationship between authentic leadership and RBSE, such that the positive effect of authentic leadership on RBSE is stronger when employee traditionality is low.

*H5*. Employee traditionality moderates the relationship between authentic leadership and employee vigor, such that the positive effect of authentic leadership on employee vigor is stronger when employee traditionality is low.

## 3 Overview of studies

We conduct two studies to test our theoretical model, including an experimental study and a field study. In Study 1, we implemented a scenario-based experiment with MBA students as subjects to examine the model, prioritizing the enhancement of internal validity and demonstrating the causal relationship between the variables. In Study 2, we conduct a multi-source and multi-wave research design in a field setting with supervisor-subordinate pairs as samples to test the model, which improves the external validity and strengthens the generalizability of findings. Notably, study 2 replicates the key findings of Study 1 after controlling for some latent variables, indicating the robustness and credibility of our results.

## 4 Study 1 experiment: method

### 4.1 Participants and main process

We recruited 260 MBA students from two universities in China given that MBA students have taken management courses and have more extensive work experience, so they can better understand experimental material and situation. Before the experiment started, we assured participants of their voluntary involvement and guaranteed the anonymity and confidentiality of their data. Then, we randomly divided participants into four equal groups, including“low authentic leadership and low employee traditionality,” “high authentic leadership and low employee traditionality,” “low authentic leadership and high employee traditionality,” “high authentic leadership and high employee traditionality”and asked them to read the materials carefully, immerse themselves into the scenarios, and answer the questions based on their true feelings. After excluding the invalid responses that failed to pass the attention check and incomplete responses, the final number of effective subjects for each condition was as follows: (1) low authentic leadership and low employee traditionality = 60; (2) high authentic leadership and low employee traditionality = 62; (3) low authentic leadership and high employee traditionality = 62; and (4) high authentic leadership and high employee traditionality = 63. Demographic characteristics of this final sample indicated that 54.7% were female, with an average age of 31.53 years.

### 4.2 Measures

In both Study 1 and 2, we adhered to the translation-back translation procedure put forward by Brislin (1970) to translate all original English scales into Chinese. All measures, unless otherwise stated, employed a five-point Likert-type scale, which ranges from 1 (strongly disagree) to 5 (strongly agree).

#### 4.2.1 Employee resilience

We measured employee resilience using the nine-item scale developed by Näswall et al. (2019). A sample item includes “Under the leadership of Manager Li, I see challenges at work as opportunities to grow” (α = 0.940).

#### 4.2.2 RBSE

We measured RBSE using the seven-item scale developed by Parker et al. (2006) and adapted it to the specific context. A sample item includes “Under the leadership of Manager Li, I am able to represent my work area in meetings with managers” (α = 0.919).

#### 4.2.3 Employee vigor

We employed the five-item scale developed by Carmeli et al. (2009) to measure employee vigor. A sample item includes “Under the leadership of Manager Li, I feel energetic at work” (α = 0.873).

#### 4.2.4 Manipulation check

We asked participants to rate their leaders in scenario experiment using sixteen-item scale developed by Walumbwa et al. (2008), with the original subject replaced accordingly. A sample item includes “Manager Li seeks feedback to improve interactions with others” (α = 0.956). Furthermore, we adopted the six-item scale developed by Farh et al. (1997) to assess whether the manipulation of employee traditionality was successful. A sample item includes “The best way to avoid mistakes is to follow the guidance of elders or leaders” (α = 0.904).

### 4.3 Experimental design

This experiment used a 2 (high vs. low authentic leadership) × 2 (high vs. low employee traditionality) between-subjects experimental design. Participants were assigned to four experimental scenarios. The experimental text consisted of two parts. The first part included a basic test primarily used to gather demographic information regarding the participants. Part two was a scenario test covering the experimental instructions, the scenario material, and related measurement items. The study scenario was set as an avatar as follows: You are an employee named “Wang Ming” in the Marketing Department of a medium-sized enterprise. In the Marketing Department, Manager Li is Wang Ming's direct supervisor. Recently, Wang Ming was assigned by Manager Li to carry out a new project, but the initial progress of the project was not going well, which made Wang Ming feel a lot of pressure. Detailed experimental materials are in Appendix. The scenario material for authentic leadership referred to the design of leadership situations by Cianci et al. (2014) The material for employee traditionality was adapted from the measurement scale developed by Farh et al. (1997) to suit the present study. Measurement items included a manipulative test of authentic leadership and employee traditionality, employee vigor, RBSE and employee resilience.

## 5 Study 1 experiment: results

### 5.1 Manipulation check

We utilized independent samples *T*-tests to evaluate the effectiveness of our manipulations for authentic leadership and traditionality. Results revealed that participants in the high authentic leadership condition scored significantly higher on the authentic leadership measure (M = 4.194, SD = 0.346) compared to those in the low authentic leadership condition (M = 2.272, SD = 0.614), *t* (245) = −30.380, *p* < 0.001, Cohen's d = −3.882. Additionally, participants in the high traditionality condition reported higher scores on the traditionality measure (M=3.784, SD=1.012) relative to the low traditionality condition (M = 2.216, SD = 0.959), t (245) = −12.493, *p* < 0.001, Cohen's d = −1.596. Thus, these findings indicated that both manipulations were successful.

### 5.2 Hypothesis testing

First, we conducted ANOVA to test the effect of authentic leadership on employee resilience. Results indicated that, M_high authentic leadership_ = 4.239, M_low authentic leadership_ = 3.704, F _(1, 243)_ = 45.781, *p* < 0.001, η^2^ = 0.157. Therefore, authentic leadership could significantly support employee resilience. Thus, hypothesis 1 was supported.

Then, we utilized bootstrapping to estimate the mediating roles of RBSE and employee vigor, generating 5,000 bootstrapped samples to compute 95% confidence intervals (CI) for indirect effects (MacKinnon et al., 2004). Results indicated that the indirect effect of authentic leadership on employee resilience via RBSE was statistically significant and positive [indirect effect = 0.084, 95%CI = (0.032, 0.158)], thus supporting hypothesis 2. Similarly, the indirect effect of authentic leadership on employee resilience via employee vigor was also significant and positive [indirect effect = 0.096, 95%CI = (0.044, 0.165)], providing support for Hypothesis 3.

**Figure 2:**
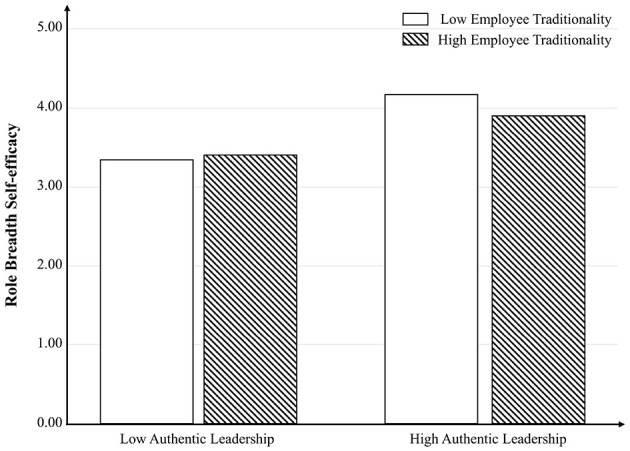
The moderating role of employee traditionality on the relationship between authentic leadership and RBSE in Study 1. The interaction effect between authentic leadership and employee traditionality on RBSE is not significant.

**Figure 3:**
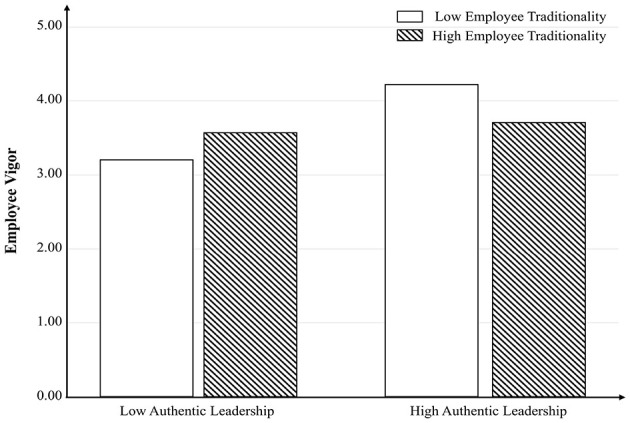
The moderating role of employee traditionality on the relationship between authentic leadership and employee vigor in Study 1. Employee traditionality negatively moderates the relationship between authentic leadership and employee vigor.

Finally, we used MANOVA to examine the moderation role of employee traditionality (Kim et al., 2001). Results revealed that the interactive effect of authentic leadership and employee traditionality did not exert a significant predictive influence on RBSE [F_(1, 243)_ = 3.017, *p* = 0.084 > 0.05]. Figure 2 was plotted to further indicate that this relationship was not significant and positive. All of these meant that Hypothesis 4 was not supported. Conversely, the interactive effect of authentic leadership and employee traditionality exerted a significant predictive effect on employee vigor [F_(1, 243)_ = 22.312, *p* < 0.001, η^2^ = 0.084]. These findings provided support for Hypothesis 5. To further clarify this relationship, Figure 3 was generated, demonstrating that the interaction effect was significant and positive specifically when employee traditionality was low.

### 5.3 Discussion

Study 1 provided initial evidence that authentic leadership could not only directly support employee resilience, but also indirectly support it by nurturing RBSE and employee vigor. In addition, lower levels of employee traditionality strengthened the positive relationship between authentic leadership and employee vigor. However, the relationship between authentic leadership and RBSE remained unaffected by the levels of employee traditionality, which was not consistent with our hypothesis. Study 1 had two main limitations. First, while the experimental design offered strong internal validity, it suffered from limited generalizability and external validity. Second, prior research on employee resilience has identified potential latent variables (e.g., age and educational background) that may influence study outcomes, yet Study 1 did not account for these factors. To address these limitations, Study 2 employed a field survey to examine the full theoretical model, aiming to enhance the generalizability of our findings and bolster external validity.

## 6 Study 2 field study: method

### 6.1 Participants, design and procedure

We employed a questionnaire survey approach and collected two waves of data through the alumni networks in China, mainly involving in the pharmaceutical company, IT company, new energy company, and machinery manufacturing. In these companies, due to higher probability of innovation failure and stronger uncertainty factors, employee resilience was more important for improving organizational performance and gaining competitive advantages. Two separate questionnaires were designed: one for employees and another for their direct supervisors. With assistance from human resource managers in the participating companies, we coded the questionnaires to pair leaders with their respective subordinates. The questionnaires were then distributed to respondents via WeChat, which is widely used as a multi-purpose messaging application in China (Qin et al., 2020). Leaders and subordinates in our survey need to be co-located, and subordinates reported directly to their leaders. The questionnaires explicitly detailed the research purpose and procedures, and assured respondents that the survey was voluntary, confidential, only for academic purposes, and free of any commercially confidential information.

In total, we sent the online questionnaires to 90 leaders and 500 subordinates. As previous research has suggested that lags of at least 1 month help reduce the common method bias (Ostroff et al., 2002). Hence, data collection was conducted at two time points with a 1 month interval. At Time 1 (T1), 500 subordinates completed measures of authentic leadership, employee vigor, RBSE, employee traditionality, and provided demographic details. At Time 2 (T2), 1 month after T1, 90 leaders evaluated their subordinates' employee resilience. Following data matching across the two waves and removal of invalid responses and missing data, the final matched sample comprised 85 leaders and 424 subordinates, resulting in response rates of 94.44% and 84.80%, respectively.

Among subordinates, 50.2% were male and 49.8% were female. Most respondents (65.6%) were under 30 years old, and 32.3% were aged 31-40. Regarding educational attainment, 80.2% of subordinates held a bachelor's degree or higher. In terms of working years, 36.8% of subordinates had worked more than 5 years. For leaders, 71.8% were male, and 28.2% were female. For age distribution, 50.6% of leaders were aged 31-40, and 29.4% were aged 41-50. Educationally, 83.6% of leaders held a bachelor's degree or higher, with an average organizational tenure of 7.1 years.

### 6.2 Measures

#### 6.2.1 Authentic leadership (T1)

We used the same scale with Study 1, which is developed by Walumbwa et al. (2008), and replaced the original subject accordingly. A typical item was “My supervisor demonstrates beliefs that are consistent with actions” (α = 0.902).

#### 6.2.2 Employee resilience (T2)

We measured employee resilience using Näswall et al. (2019) scale as in Study 1 (α = 0.925).

#### 6.2.3 RBSE (T1)

We measured RBSE using Parker et al. (2006) scale as in Study 1. Participants were asked to rate their confidence in performing various tasks, such as “How confident you feel about making a presentation in front of many colleagues” (α = 0.909).

#### 6.2.4 Employee vigor (T1)

We measured employee vigor using Carmeli et al. (2009) scale as in Study 1 (α = 0.860).

#### 6.2.5 Employee traditionality (T1)

We measured employee traditionality using Farh et al. (1997) scale as in Study 1 (α = 0.853).

#### 6.2.6 Control variables

Following prior research on leader-employee relationship and employee resilience (Hirak et al., 2012; Caniëls and Baaten, 2019), we controlled for subordinates' age, gender, education level and organizational attributes in our study. Subordinates' age, gender and education level are associated with accumulated knowledge, innovation ability, problem-solving ability and social network, which could have an impact on how employees cope with challenges and difficulties (Lu et al., 2019; Wadei et al., 2021). Organizational attributes was controlled because companies with different attributes have great differences in values, management styles, human resource strategies and so on, which will affect employees' knowledge and resource acquisition, attitudes and values, and also lead to differences in employees' behaviors, especially in a crisis (Edú-Valsania et al., 2016).

## 7 Study 2 field study: results

### 7.1 Preliminary analysis

Prior to testing the hypotheses, we conducted a series of confirmatory factor analyses (CFAs) using Mplus 8.3 to assess the discriminant validity between our key variables. The results were shown in Table 1. Considering that authentic leadership scale comprised numerous measurement items and spanned four dimensions, which could cause parameter instability and increase standards errors, in line with previous research (Ou et al., 2014), we used item parceling to generate four parcels for authentic leadership according to its four dimensions. As presented in Table 1, the hypothesized five-factor model (*x*^2^/df = 1.377, RMSEA = 0.030, CFI = 0.978, TLI = 0.976) demonstrated superior fit compared to alternative models, indicating satisfactory discriminant validity among our key variables. Notably, employee resilience was assessed by leaders at Time 2 (T2), whereas all other variables were obtained from employee self-reports, which could potentially introduce common method bias. To address this, following the suggestion of Podsakoff et al. (2003), we constructed a six-factor model by adding a common method factor to the five-factor model. As presented in Table 1, although the six-factor model fit slightly better than the five-factor model, the differences in fit indices were not statistically significant, suggesting that all variables in the study were distinguishable.

**Table 1:** Confirmatory factor analyses results in study 2.

**Model**	***χ^2^***	***df***	***χ^2^*/*df***	**RMSEA**	**SRMR**	**CFI**	**TLI**
Six factors model (AL, EV, RBSE, ER, ET, CMV)	543.163	394	1.379	0.030	0.035	0.978	0.976
Five factors model (AL, EV, RBSE, ER, ET)	543.772	395	1.377	0.030	0.035	0.978	0.976
Four factors model (AL + EV, RBSE, ER, ET)	891.458	399	2.234	0.054	0.053	0.927	0.920
Three factors model (AL + RBSE + EV, ER, ET)	1322.378	402	3.289	0.073	0.062	0.864	0.853
Two factors model (AL + RBSE + EV + ER, ET)	2331.851	404	5.772	0.106	0.087	0.714	0.693
Single factor model (AL + RBSE + EV + ER + ET)	3160.571	405	7.804	0.127	0.115	0.592	0.562

AL, authentic leadership; EV, employee vigor; RBSE, Role breadth self-efficacy; ER, employee resilience; ET, employee traditionality; CMV, common factor; “+” represents the combination of factors.

The means, standard deviations, reliabilities and correlations among the variables are presented in Table 2.

**Table 2:** Means, standard deviations, and correlations among variables in study 2.

**Variable**	***M***	***SD***	**1**	**2**	**3**	**4**	**5**	**6**	**7**	**8**
1. Gender	1.498	0.501								
2. Age	1.368	0.534	−0.076							
3. Education	1.927	0.573	0.053	0.320^**^						
4. Nature	1.625	0.485	0.001	0.078	0.259^**^					
5. AL	3.759	0.639	0.031	0.171^**^	0.156^**^	0.017				
6. EV	3.702	0.779	0.008	0.200^**^	0.130^**^	−0.036	0.430^**^			
7. RBSE	3.730	0.735	−0.053	0.235^**^	0.117^*^	0.009	0.503^**^	0.536^**^		
8. ET	2.992	1.004	0.165^**^	−0.153^**^	−0.116^*^	−0.111^*^	−0.079	−0.127^**^	−0.117^*^	
9. ER	3.992	0.617	0.011	0.172^**^	0.030	0.000	0.445^**^	0.489^**^	0.515^**^	−0.118^*^

^*^p<*0.05*, ^**^p<*0.01*. Nature, nature of the company; AL, authentic leadership; EV, employee vigor; RBSE, Role breadth self-efficacy; ET, employee traditionality; ER, employee resilience.

### 7.2 Hypothesis testing

We utilized Mplus 8.3 to establish a structural equation model for hypothesis test, with results presented in Table 3. In Model 3, after controlling for demographic variables and organizational attributes, authentic leadership exhibited a statistically significant positive effect on employee resilience (β = 0.583, *p* < 0.01), supporting Hypothesis 1. Specifically, Model 1 revealed a positive relationship between authentic leadership and RBSE (β = 0.619, *p* < 0.01). Model 2 further showed that authentic leadership was positively associated with employee vigor (β = 0.548, *p* < 0.01). Notably, when authentic leadership, RBSE, and employee vigor were simultaneously included in Model 4 to predict employee resilience, the direct effect of authentic leadership on employee resilience diminished from β = 0.583(*p* < 0.01) to β = 0.225(*p* < 0.05). This reduction, coupled with significant indirect effects through RBSE (β = 0.280, *p* < 0.01) and employee vigor (β = 0.256, *p* < 0.01), provided evidence for their mediating roles between authentic leadership and employee resilience. Thus, Hypotheses 2 and 3 were supported.

**Table 3:** The results of main effect and mediating effect in study 2.

**Variable**	**RBSE**	**EV**	**ER**	
	**Model 1**	**Model 2**	**Model 3**	**Model 4**
Gender	0.004	−0.062	0.019	0.032
Age	0.116^*^	0.156^**^	0.103^*^	0.055
Education	0.025	−0.007	−0.090	−0.090
Nature	−0.062	−0.012	0.015	0.030
AL	0.619^**^	0.548^**^	0.583^**^	0.225^*^
RBSE				0.280^**^
EV				0.256^**^
*R* ^2^	0.412^**^	0.319^**^	0.352^**^	0.398^**^

^*^p < 0.05, ^**^p < 0.01. Nature, nature of the company; AL, authentic leadership; RBSE, Role breadth self-efficacy; EV, employee vigor; ER, employee resilience.

To further validate the mediating roles of RBSE and employee vigor, we used bootstrapping with 5,000 resamples for robust test. The results revealed that the indirect effect mediated by RBSE was statistically significant [indirect effect = 0.173, 95%CI = (0.058, 0.305), not containing 0]. Hypothesis 2 was further verified. Likewise, the indirect effect mediated by employee vigor was also statistically significant [indirect effect = 0.140, 95%CI = (0.053, 0.234), not containing 0], further supporting Hypothesis 3.

**Figure 4:**
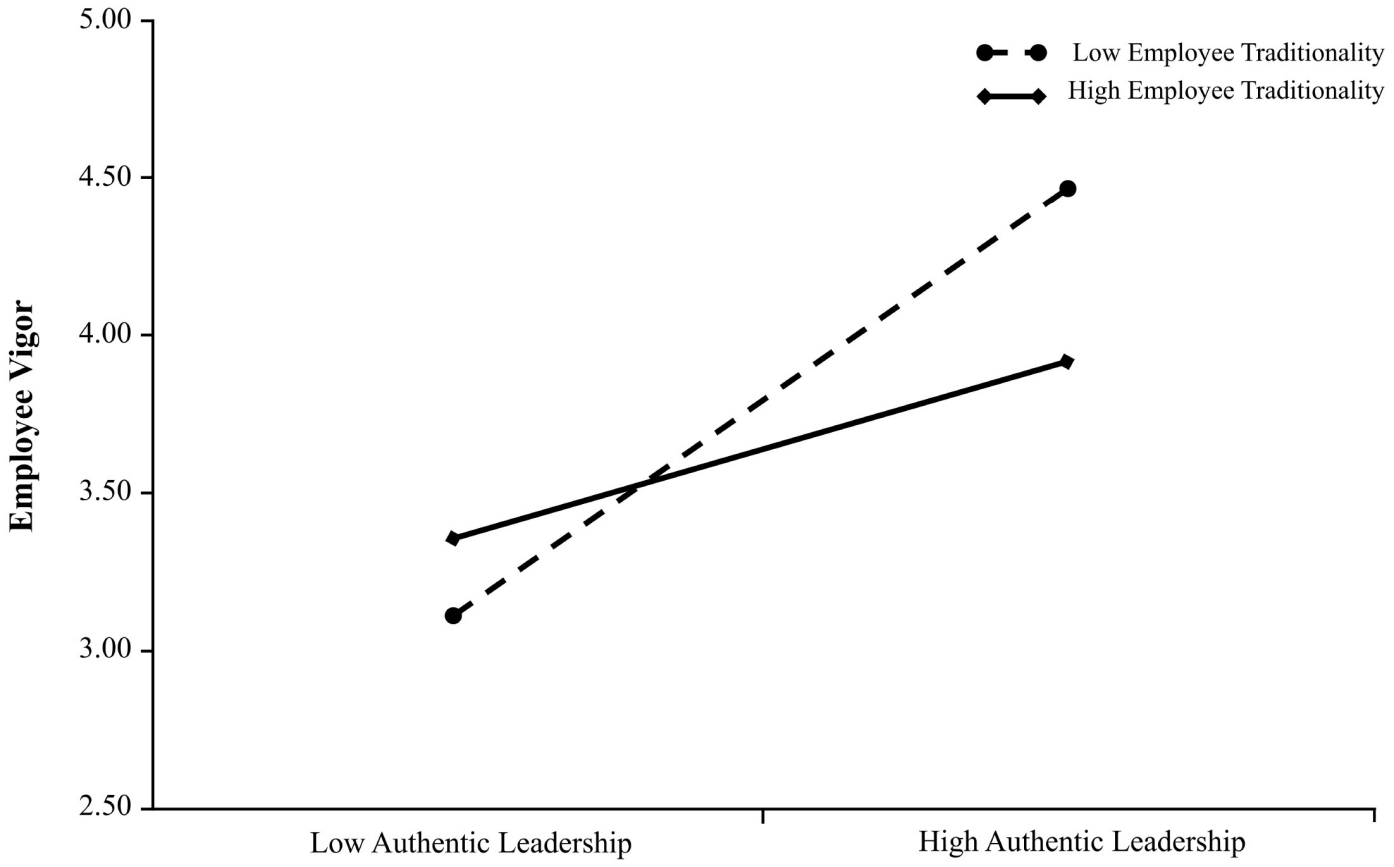
The moderating role of employee traditionality on the relationship between authentic leadership and employee vigor in Study 2. The positive association between authentic leadership and employee vigor is stronger when employee traditionality is low rather than high.

Table 4 displays the moderation role of employee traditionality. In Model 2, the interaction term between authentic leadership and employee traditionality did not significantly predict employee resilience (*p* > 0.05), which didn't provide supports for Hypothesis 4. In Model 4, however, the interaction term of authentic leadership and employee traditionality exerted a significant negative effect on employee vigor (β = −0.192, *p* < 0.01). This suggests that the positive relationship between authentic leadership and employee vigor was more pronounced when employee traditionality was low compared to when it was high, thereby supporting Hypothesis 5. Figure 4 visually illustrates this moderating effect.

**Table 4:** The moderating effect analysis of employee traditionality in study 2.

**Variable**	**RBSE**		**EV**	
	**Model 1**	**Model 2**	**Model 3**	**Model 4**
Gender	−0.058	−0.061	0.022	0.015
Age	0.132^**^	0.131^*^	0.085	0.107^*^
Education	−0.007	−0.009	0.023	0.027
Nature	−0.013	−0.007	−0.069	−0.056
AL	0.644^**^	0.635^**^	0.564^**^	0.470^**^
ET	−0.046	−0.036	−0.098	−0.080
AL × ET		−0.077		−0.192^**^
R^2^	0.444^**^	0.436^**^	0.350^**^	0.287^**^

^*^p < 0.05, ^**^p < 0.01. Nature=nature of the company, AL=authentic leadership, ET=employee traditionality, RBSE=Role breadth self-efficacy, EV=employee vigor.

### 7.3 Discussion

Study 2 not only replicated the key findings of Study 1, but also extended the external validity of the overarching research. These consistent results further underscore the critical role of the cognitive-affective mechanism in explaining the relationship between authentic leadership and employee resilience. Additionally, our analysis further demonstrated that employee traditionality only moderated the link between authentic leadership and employee vigor, failing to influence the relationship between authentic leadership and RBSE. This pattern of results underscores the distinct impacts of individual cultural traits on different psychological and behavioral pathways.

## 8 General discussion

Drawing on CAPS theory, our research investigates the relationship between authentic leadership and employee resilience. It highlights the dual mechanisms of RBSE (cognitive mechanism) and employee vigor (affective mechanism) to elucidate how authentic leadership influences employee resilience, as well as the moderating role of employee traditionality. The findings from two studies (including 247 samples in a scenario experiment and 85 leaders-424 subordinates pairs in a field study) provide consistent evidence supporting that authentic leadership not only exerts a significant positive direct impact on employee resilience, but also indirectly supports it by promoting RBSE and nurturing employee vigor. Moreover, employee traditionality is found to negatively moderate the relationship between authentic leadership and employee vigor, which means that lower levels of employee traditionality amplifies this effect. Notably, however, employee traditionality does not moderate the link between authentic leadership and RBSE, which diverges from our hypothesis. The possible reason is that RBSE represents a cognitive response, and compared with strong and unstable emotional responses, individual cognitive responses tend to be more rational (Mischel and Shoda, 1995). Specifically, employees with higher traditionality, who comply with leadership authority and emphasize social hierarchy and role obligations, allocate additional cognitive resources to meet the requirements of authentic leadership, even for tasks beyond their prescribed duties, thereby facilitating the formation of RBSE. Conversely, employees with lower traditionality, characterized by a greater emphasis on fair and equal relationships between leaders and subordinates, identify with authentic leadership's behaviors and are willing to fully mobilize their cognitive resources to accomplish organizational tasks, which also contributes to RBSE. This implies that individuals with different levels of traditionality, despite variations in their cognitive approaches, ultimately converge in their responses to authentic leadership. By systematically testing the theoretical model across both studies, this research thoroughly explores the underlying mechanisms and boundary conditions of authentic leadership on employee resilience, offering actionable insights for organizations aiming to enhance employee resilience.

### 8.1 Theoretical implications

Our study offers three primary theoretical contributions.

First, we uncover a dual-mediated pathway through which authentic leadership influences employee resilience, centered on cognitive (RBSE) and affective (employee vigor) mechanisms. This advances existing literature by identifying a previously unexplored integrative mechanism. Prior research on the impacts of leadership on employee resilience has mostly focused on a single perspective, such as employees' psychological process or cognitive process (Li and Zhang, 2022; Mao et al., 2022), ignoring the joint effect of cognitive factors and affective factors. By adopting a cognitive-affective dual perspective, our work not only deepens understanding of how authentic leadership shapes employee resilience, but also provides a novel theoretical perspective to explain how leaders' behaviors translate into subordinates' responses.

Second, we identify the moderating role of employee traditionality, addressing recent calls to integrate cultural factors into leadership research (Cheng et al., 2021). Scholars have long underscored the significance of cultural contingency in examining leadership effectiveness (Spreitzer et al., 2005; Yang et al., 2017; Shen et al., 2019). However, limited research has explored how specific cultural traits moderate the effects of authentic leadership. Given this, we explore the moderating effects of employee traditionality, a crucial cultural value variable in China, extending the current knowledge on moderating the influences of authentic leadership and highlighting the need for future research to attend to cultural contingencies in leadership processes.

Finally, grounded in the CAPS theory, we integrate authentic leadership as an external situational factor, RBSE as a cognitive factor, employee vigor as an emotional factor and employee traditionality as individual difference into a unified framework to explain employee resilience, which not only provides a more systematic and comprehensive theoretical framework to understand how authentic leadership supports employee resilience, but also enriches the literature on CAPS theory and broadens its application range.

### 8.2 Practical implications

Our study yields three key practical implications.

First, our results underscore the importance of recruiting and developing leaders who demonstrate authentic and positive behaviors within organizations. Nowadays, the performance and competency of leaders are widely concerned (Cheng et al., 2021). However, their positive psychological capacities and construction of a satisfying working atmosphere are often overlooked, especially in tough times. Our study demonstrates the efficacy of authentic leadership in motivating employees to resist pressure and difficulties in challenging environment. Hence, enterprises can set up some training courses and programs to help leaders learn and embody authentic leadership principles in their daily practices.

Second, prioritizing employees' cognitive and emotional wellbeing is critical. On one hand, leaders should demonstrate care and respect for subordinates, foster a positive work atmosphere, and encourage open communication and innovation. Such efforts not only boost employees' confidence in proposing new ideas but also enhance their willingness to take on new tasks. On the other hand, leaders can increase employees' positive emotions by means of concern, praise and encouragement, and help subordinates understand and use their own advantages to improve employee vigor and maintain a good work state.

Third, our findings on the moderating role of traditionality suggest that leaders should attend to variations in employees‘ traditionality levels, especially within Chinese contexts, and adopt differentiated strategies accordingly. For employees with high traditionality, leaders may foster vigor through alternative behaviors, such as emphasizing equality, encouraging self-expression, and supporting employees' ideas and professional development. For those with low traditionality, leaders should prioritize demonstrating authentic leadership behaviors to ignite vigor and enthusiasm at work.

### 8.3 Limitations and directions for future research

While our study offers several strengths, it is not without limitations that warrant attention in future research.

First, our study explored how authentic leadership supported employee resilience based on CAPS theory. Although the antecedents of employee resilience have been enriched to a certain extent, there are still many other theories which can explain the proposed model and different leadership types on employee resilience that need to be further discussed. For instance, future research might explore these relationships from the perspectives of conservation of resources theory or examine the differential effects of other leadership styles (e.g., spiritual leadership) on employee resilience. Expanding the theoretical scope would deepen our comprehension of the mechanisms underlying authentic leadership's impact.

Second, in this study, the data we collected were from China within a single cultural context, implying that the findings may be moderated by cultural factors besides employee traditionality, such as high power distance and collectivist orientation. This may raise concerns about external validity and potentially constrain the generalizability of our findings to other regions and populations, especially in countries that emphasize individualism orientation. Therefore, future research can expand the scope of sample sources or reset our model in different cultural backgrounds or different countries and discuss the potential cross-cultural implications.

Third, the data for Study 2 were primarily collected from companies in the pharmaceutical, IT, new energy, and machinery manufacturing sectors. In these companies, employees tend to be younger and have a higher level of education. Their work styles and core values may differ from those of older or less-educated employees, which could limit the external validity of the findings. Future research could improve generalizability by increasing the sample proportion of older employees and individuals with diverse educational backgrounds from a wider range of industries.

Fourth, this study measured authentic leadership using the scale developed by Walumbwa et al. (2008). Although this scale has been widely adopted and demonstrates good validity, existing research has also noted its potential limitations, such as restricted cross-cultural generalizability and risks associated with common method bias. Future studies could employ interviews or case studies to further validate the measurement validity of authentic leadership.

Fifth, this study adopted an experimental study and a field study to test the theoretical model. Study1 measured employee traditionality in specific contexts through contextualized manipulations, while Study 2 assessed employee traditionality using a standardized scale. The results of the two studies were complementary, but differences in operation, response patterns, and cognitive load between the methods may lead to measurement nonequivalence for employee traditionality as a cultural construct across approaches. Future research could conduct both scenario-based experiments and filed studies within the same population or employ longitudinal tracking methods to enhance the validity of the findings.

## Data Availability

The raw data supporting the conclusions of this article will be made available by the authors, without undue reservation.
